# The Impact of the COVID-19 Pandemic on Lifestyle and Wellbeing of Children, Adolescents and Their Parents: A Qualitative Study

**DOI:** 10.3390/children9121929

**Published:** 2022-12-08

**Authors:** Kelly G. H. van de Pas, Marijn L. Hesselink, Robin Schlechtriem, Lisanne Arayess, Johanna M. Kreutz, Anita C. E. Vreugdenhil

**Affiliations:** 1 Centre for Overweight Adolescent and Children’s Healthcare (COACH), Department of Pediatrics, Maastricht University Medical Centre, 6229 HX Maastricht, The Netherlands; 2 Department of Surgery, Máxima Medical Center, 5504 DB Veldhoven, The Netherlands; 3 NUTRIM School of Nutrition and Translational Research in Metabolism, Maastricht University, 6229 ER Maastricht, The Netherlands; 4 Department of Pediatrics, Maastricht University Medical Centre+, 6229 HX Maastricht, The Netherlands

**Keywords:** COVID-19, pandemic, semi-structured interviews, lifestyle, wellbeing, children, adolescents

## Abstract

Prior studies have shown that changes in daily structure and habits due to the COVID-19 pandemic affected the lifestyle and wellbeing of families. This study aimed to obtain in-depth information on children’s and adolescents’ experiences regarding their lifestyle and wellbeing during the pandemic. Semi-structured interviews with fifteen families were carried out between May and November 2021. Directed content analysis was used to analyze the transcripts and fundamental qualitative description to describe the results. Children and adolescents revealed an overall unhealthier lifestyle and decreased wellbeing. These negative effects were even larger in adolescents and children with overweight or psychosocial complaints. Our results revealed that parents were actively involved in maintaining a normal daily structure. Furthermore, diet changes were inconsistent and dependent on food availability. An increase in screen time was experienced as inevitable, and external influences were necessary to keep children and adolescents active. Almost no effects were reported on physical health, whereas negative emotions were experienced in varying degrees. Moreover, the decrease in social interactions was reported as the most negative effect of the pandemic. The above-mentioned insights may contribute to the development of preventive measures to promote a healthy lifestyle and wellbeing of children and adolescents during future pandemics.

## 1. Introduction

In March 2020 the World Health Organization declared SARS-CoV-2 (COVID-19) as a global pandemic. Since then, national measures to prevent the spread of COVID-19 in the Dutch population have radically changed the lives of children and adolescents in the Netherlands. Governmental regulations were continuously adapted to lower the spread of COVID-19 infections and ease the burden on the healthcare system. These regulations included the closure of sport facilities, public spaces, non-essential shops, restaurants and schools [1]. Since the start of the pandemic, the regulations of schools and sport facilities for children varied from being completely closed to being open when adhering to certain rules. Examples were quarantining when a certain number of infections within a classroom were reached, not allowing sport competitions between clubs and sport facilities with restricted opening hours. Besides this, parents were regularly required to work from home and additionally had to support their children during home schooling. Social distancing further affected the support systems of families as it was advised to limit contact with people outside of the household.

Previous research has shown that changes in daily structure and habits are expected to affect the lifestyle and wellbeing of children, adolescents and their parents. The alterations in daily structure and habits due to governmental regulations during the COVID-19 pandemic resulted in weight gain, increased fatigue, decreased physical activity, increased sedentary time and an increase in mental health problems [2,3,4,5,6,7,8]. Interview studies with families in the United States of America and Indonesia revealed similar results and additionally showed that parents played a role in managing the screen time of children and promoting activity during the pandemic [9,10]. Furthermore, a systematic review reported that children and adolescents are more likely to experience high rates of anxiety and depression during and after a pandemic [11].

A Dutch qualitative mixed methods study among children with severe obesity observed an increased demand for food and attributed this to a loss of daily structure, increased stress and boredom. In this study, lower physical activity due to anxiety about COVID-19 infection was also mentioned. Besides this, parents reported deteriorated emotional wellbeing of their children due to an increase in conflicts, as the whole family was at home all the time [12]. Since these studies only included children with severe obesity who were undergoing treatment for their weight or were conducted in a different country, it is unknown whether the general population of Dutch children and adolescents also encountered these barriers. Therefore, the aim of our study is to obtain in-depth information on the experiences regarding lifestyle and wellbeing of Dutch children, adolescents and their families during the COVID-19 pandemic using semi-structured interviews.

## 2. Materials and Methods

### 2.1. Setting

Families were selected from the COLC study (Children, Obesity and Lifestyle during COVID-19). The COLC study was a national prospective digital questionnaire study concerning the lifestyle and wellbeing of Dutch children and adolescents during the COVID-19 pandemic. The study was conducted in accordance with the Declaration of Helsinki, approved by the Medical Ethical Committee of the Maastricht University Medical Centre (METC2020-1330-A-1) and registered at clinicaltrials.gov (NCT04411511). Informed consent of parents and children aged ≥12 years was obtained prior to the start of the study. Families had the opportunity to give additional informed consent to participate in interviews.

### 2.2. Participants

Recruitment of families took place via social media, traditional media, primary and secondary schools. Children aged between four and eighteen years living in the Netherlands during the COVID-19 pandemic were considered for inclusion in this study. Families who participated in the COLC study and gave additional informed consent for an interview were invited by email (*n* = 262). From May 2021 until November 2021, 25 families responded and after interviewing the first 15 families the researchers collaboratively decided that data saturation was reached. The other 10 families either did not respond to make an appointment or were only available after data saturation was reached.

All the interviewed families had Dutch nationality. During three interviews, both mother and father were present, in one case only the father, and in another case only the adolescent attended the interview. In all the other interviews, only the mother and the children were present. In seven interviews, siblings also participated next to the main child. The characteristics of the included main participants and their families are presented in Table 1. The majority of the children and adolescents had a healthy weight and reported no weight gain during the pandemic. Three adolescents stated that they had gained weight during the pandemic and these were the ones with overweight or severe obesity.

### 2.3. Procedures

Semi-structured interviews of 50 to 70 min were carried out between May 2021 and November 2021 by two female medical students (R.S. and F.B.). There was no relation between the participants and the interviewees prior to the start of the study and the main goal of the research was explained before the start of the interviews. The interviews were conducted via Microsoft Teams, recorded with a digital audio recorder and supervised by three PhD students (K.G.H.v.d.P., M.L.H. and J.M.K.). The interviews covered the topics of lifestyle and wellbeing. The interview guide is presented in Supplementary File S1 and the topics in the guide were based on the questionnaires regarding lifestyle used in the COLC study and a validated questionnaire on quality of life (KIDSCREEN-27) [16]. After a pilot interview and the first two interviews, the interview guide was adjusted to make sure that all relevant topics were covered. The interviews were conducted with all family members simultaneously. The questions were directed towards one child or adolescent as the main participant to gain insight into their experiences during the COVID-19 pandemic. Additionally, parents, and when applicable siblings, were invited to elaborate on the answers of the participating child. The interviews were semi-structured, so that flexibility and responsiveness could be guaranteed whilst still keeping the interview on track. During the interviews field notes regarding non-visual communication and important topics were made. Purposive sampling of children with overweight and obesity was used to ensure that a variety of answers were given, since the majority of the COLC participants had a normal weight. Families were included until no new insights were discovered during the interviews. The Consolidated criteria for Reporting Qualitative research (COREQ) were used as the reporting guidelines [17].

### 2.4. Data Analysis

The audio recordings of the interviews were manually transcribed, removing stutters and repetitions, finally resulting in verbatim transcriptions. The transcripts were not returned to the participants for comments or corrections. All transcripts were examined line-by-line and directed content analysis was used to analyze the transcripts [18]. The themes and categories were identified in advance and based on the same topics covered in the interview. The themes were lifestyle and wellbeing: lifestyle was divided into three categories (daily structure, diet and physical activity), as was wellbeing (physical health, mental health and social life). The transcripts were individually coded using Nvivo 14 (version 20.4.0, QSR International Pty Ltd.) by researchers K.G.H.v.d.P. and R.S. The coding was done according to the predefined categories and supplemented with subcategories that came up during the coding (Supplementary File S2). The codes of the transcripts were compared; in case of differences, the code was discussed until an agreement between researchers was reached. Within the categories K.G.H.v.d.P. and M.L.H. analyzed the codes, looking for patterns and answers that were notably different from the overall opinion. Fundamental qualitative description was used to describe the results [19]. Quotes that best reflected this were identified by K.G.H.v.d.P. and M.L.H. and verified by the rest of the research team. In the final stage, the quotes were translated from Dutch to English.

## 3. Results

### 3.1. Lifestyle

#### 3.1.1. Families Were Actively Involved in Maintaining a Normal Daily Structure—Daily Structure

The COVID-19 pandemic influenced the daily structure of the interviewed families. However, the majority of the families felt that their daily routine had almost stayed the same. Parents actively tried to keep the daily structure of their children the same as before the pandemic.

“I think I am trying to live normal (daily structure comparable to the period before the pandemic) with them”

COLC15 (mother)

One mother indicated that she maintained a daily structure comparable to the period before the pandemic to improve the wellbeing of her children.

“Well, they are less irritable now (when maintaining a normal daily structure). And less likely to get angry… It’s not just hard for adults but for children too”

COLC01 (mother)

Other reported facilitators for maintaining a daily structure were schools that provided online classes, as children and adolescents had to get up early just like on regular schooldays, and other external factors such as the work of parents and extracurricular care. Notably, maintaining a daily structure seemed harder for adolescents and youth with overweight or psychosocial complaints. These older and vulnerable children more often reported changes in daily structure.

“I just went online (for 10 min of school instructions) and after that, I shut down the laptop and I went back to bed, back to sleep again”

COLC13 (adolescent)

With regard to sleeping times, parents mentioned that it is hard to define if the sleeping times altered due to the COVID-19 pandemic or due to their children’s aging.

“I think he’s allowed to go to bed a little later, but would have been able to otherwise, that it’s not specifically because of COVID-19”

COLC02 (father)

Other children, adolescents and parents reported that sleeping times shifted due to school starting later and less time spent commuting to school, but that the total sleep duration did not change.

“I think we all went to sleep later. And I do get up later because those classes, they start a little bit later…”

COLC04 (adolescent)

Adolescents often mentioned that their sleeping times altered, which might be due to less parental influence in older children. On the contrary, the nature of the child is also important as one adolescent reported that he installed an app on his phone so that he could no longer access his phone after a certain time.

“Yes, I did notice… that especially late, right before bed, I was on my phone and that that also had an effect on how fast I could fall asleep.”

COLC04 (adolescent)

#### 3.1.2. Changes in Diet Were Dependent on Food Availability—Diet

The COVID-19 pandemic changed the diet and eating behavior of the interviewed families. However, changes were inconsistent. Some families mentioned making healthier choices while other families indicated that snacking behavior of their children increased. Examples of healthier food choices were that children ate more fruit at home than they would at school. Some families mentioned they did groceries locally and cooked dinners at home more often than before the pandemic. Besides this, families reported having a luxurious lunch together more frequently than before the pandemic and this improved family bonding time. However, the products consumed during these lunch breaks were less healthy and children stated that they ate more than they would at school.

“(Did your lunch change?) Yes, she had more waffles, grilled cheese and eggs for lunch. It became more like different, less healthy, and at home you can make it fresh”

COLC05 (mother)

The main determinant in the choice for healthy or unhealthy food and the amount of food was the availability and the easy access of food at home. Families that experienced an increased workload due to the COVID-19 pandemic, for example health care workers and schoolteachers, indicated that they tended to make more easy and fast choices. This led to getting more takeout or ready meals for dinner. Families also mentioned that children more frequently asked for snacks.

“Well, I think there were certainly more requests for sweets”

COLC06 (mother)

The children themselves indicated that this was out of boredom and easier availability compared to at school. The possibility that children were hungrier because they spent more time playing outside was also mentioned by some families.

“But I also think it’s because I have nothing to do at home and at school I don’t really think about food, then I’m busy with other things. But at home yes, I generally eat a lot. Because at home, I can always grab something and at school, I only eat during the breaks”

COLC05 (adolescent)

Adolescents indicated that they couldn’t go to the supermarket with friends during school hours as the schools had closed down. This could have led to buying fewer unhealthy food products. At the same time, they mentioned that they went to get more takeout fast-food as a way of socializing with friends.

“Now, after I had dinner I would go to McDonald’s with a friend, there are many other friends and that was the way of seeing them”

COLC13 (adolescent)

Notably, a few families mentioned that the father had an unhealthy influence on the groceries that were bought and on the foods that were consumed, which happened due to the father being at home more.

“(speaking to child) When your father did the groceries there was more junk food available at home”

COLC11 (mother)

Most families concluded that the positive effects of the COVID-19 pandemic on their diet and food behavior diminished as soon as life went back to how it was before the pandemic. Contrastingly, unhealthy behaviors that started during the COVID-19 pandemic were more difficult to reverse and sometimes remained.

“The structure in my eating pattern is lost. And that still is the case”

COLC14 (adolescent)

#### 3.1.3. The Increase in Screen Time Was Experienced as Inevitable—Screen Time

According to children and adolescents their screen time has inevitably increased during the COVID-19 pandemic. Determinants for the increase in screen time were school, because lessons were offered online, and boredom due to fewer activities. Furthermore, it was reported as the only way to connect with friends, especially for older children.

“The only way of talking to your friends or like anyone that doesn’t live with you was via the computer so I guess it only makes sense that you would use the computer a lot more than we used to. Because that’s the only connection to the outside world”

COLC12 (adolescent)

Some parents also mentioned that they used screens as pastimes or as an easy distraction for their children and adolescents when parents wanted to work, had online meetings or were ill.

“Well, during COVID-19 you (children) definitely watched more screens, because if we had an important meeting… Then you had to be quiet”

COLC 06 (mother)

Besides this, the role of schools in limiting screen time diminished during the pandemic. Instead of going to school and participating in physical classes, schools provided online classes.

While most families reported an increase in screen time, some reported that the screen time of their children stayed the same. However, upon further questioning, screen time turned out to be more than before the pandemic, suggesting that it is difficult for families to make an adequate estimation of their screen time during the pandemic.

#### 3.1.4. Keeping Children and Adolescents Active Requires External Factors—Physical Activity

The COVID-19 pandemic influenced the physical activity of children and adolescents. However, these changes were inconsistent as younger children reported playing outside more, whereas adolescents did not. External factors such as the organization of alternative activities or the influence of the parents were important for keeping the children active.

At the beginning of the pandemic, all sports activities were cancelled and children and adolescents did not have physical education at school. Additionally, they did not have to cycle or walk to school, possibly leading to a further decline in physical activity. Physical activity reduction resulted in boredom and fewer social interactions among participants. Some children and adolescents also mentioned that they could not release their energy due to this.

(Why did you miss sports?) “Then you are with some people from your team again and you learn a little bit. So the fun and you have also exercised. You can release your energy and I like that”

COLC04 (adolescent)

Notably, a couple of children and adolescents said that they missed the competitions the most.

“I missed going to the matches actually. Like playing in a match”

COLC12 (adolescent)

As the pandemic proceeded, alternative activities were organized by sport clubs, schools or parents to keep the children and adolescents active. One family bought a trampoline, whereas others tried to do sports, go on a walk or cycle together as a family. However, it was not always easy to motivate child, and it seemed from our observations that younger children were more approachable for this.

“But it is not very easy when you have been sitting inside together all day, come on kids we are going to do something fun again (playing tennis). Then it is not like they are always cheering of what a good idea”

COLC04 (mother)

Furthermore, only one adolescent reported that she sought other fulfillment for the reduced physical activity by dancing in her room with her sister.

During the pandemic, the majority of the families mentioned that their children played outside more. This increased due to fewer school hours, no other organized activities and the generally good weather.

“You finish school earlier and then you can play outside for longer. Or you just play outside when otherwise you could not, for example field hockey was canceled and instead of this you can play outside”

COLC 03 (adolescent)

It seemed that the children and adolescents who already played outside before the pandemic spent more time playing outside. In line with this, the children who did not play outside before the pandemic did not spend more time playing outside. This was particularly the case in older children. A barrier against playing outside was social control in the neighborhood. Some neighbors did not want children and adolescents playing outside due to the fear of spreading COVID-19.

“Because the people in our community were more afraid (of COVID-19), we just did not let the kids play together”

COLC10 (mother)

The important findings with regard to the lifestyle of families during the pandemic are summarized in Table 2.

### 3.2. Wellbeing

#### 3.2.1. Experienced Negative Effects of COVID-19 on Physical Health Were Limited—Physical Health

Overall, children and adolescents considered their physical health as good. They did not experience significant differences because of the COVID-19 pandemic. Despite that, almost all children indicated that if they had a cold or any symptoms that could be related to a COVID-19 infection, the effect was more negative than before. They were not allowed to go to school without testing and felt restricted by this.

“Because normally when you have a cold you can just go to school and now they had to stay at home”

COLC08 (mother)

Besides this, some feared the impact a COVID-19 infection could have on the health of their elderly family members. Energy levels were reported generally to be similar, compared to before the pandemic, with some exceptions.

“Well in my opinion, since the start of the pandemic, you are a little, how to say, more lethargic, like if you are in such a state it is harder for me to get you off the couch”

COLC04 (mother)

One adolescent also indicated that his endurance was lower after he was infected with COVID-19 and that it took a while to regain his fitness.

“After that, you really have to rebuild your condition because of fatigue. And the first time when I was allowed to go back to soccer training, I ran from one side of the penalty area to the other side and I was completely exhausted”

COLC08 (adolescent)

#### 3.2.2. Feelings of Irritability, Anger, Loneliness and Sadness Were Experienced in Varying Degrees—Mental Health

In general, the children and adolescents were happy and experienced good mental health. However, almost all interviewed families felt that the mental health of their children and adolescents decreased during the pandemic and it decreased even more in adolescents and vulnerable children, e.g., the ones with overweight or psychosocial complaints.

Feelings of irritability, anger, loneliness, sadness, boredom or fear were experienced by almost all children and adolescents, although to varying degrees. Some children mentioned that these feelings were due to a lot of time spent with the same people in the same environment and less social contact.

“I sometimes have moments when I’m just a little angry, but that’s… because all the time when you are home you see you guys (points at father) all day”

COLC02 (child)

“Especially that I could not play with my friends, you just could not go on playdates during the pandemic”

COLC08 (child)

Others reported boredom as a result of fewer challenges, particularly in school. Notably, one mother indicated that her children learned to become more resilient to alterations and disappointments due to the continued cancellation of activities.

“It was an accumulation of cancelled activities. Then you have to show some resilience as a kid”

COLC03 (mother)

One adolescent with severe obesity reported that she was depressed. She was already depressed before the pandemic and her symptoms worsened due to it. For her, it was a struggle to get through each day.

“Normally you go to school and have social contacts around you anyway. And as soon as that is no longer allowed, that you cannot go to school and that your social contacts are taken away from you. Then your depressive phase goes from a normal depression to a more extreme one”

COLC13 (adolescent)

Two other families also reported that one of their children had a hard time during the pandemic, e.g., depressive symptoms and excessive worrying. They mentioned that the problems of their children were signaled by the school and that they received help.

Interestingly, when asking children, adolescents and their families about their mental health, the majority attenuated the consequences of the pandemic. They reported that in their case it was not so bad and felt that it was worse in other families.

“I did not like it (COVID-19), but I don’t like complaining about it seeing that there are people who had it so much worse”

COLC12 (adolescent)

#### 3.2.3. Loss of Social Interactions Was Experienced as the Most Negative Effect of COVID-19—Social Life

Children and adolescents reported that they had fewer social interactions with friends. The majority stated that this was the most negative effect of the pandemic. Children who played outside indicated they made new friends and met with them more often than before, mainly because they had more time as they spent less time at school. Participants who stayed inside more found it harder to make new friends. It appeared that it was more difficult to arrange meetings with friends without the contact moments at school.

“Previously (before the pandemic) I did not meet that often with friend(s) outside of school, so to not meet up with them and to not see each other at school or at the sports club, yes that was hard”

COLC04 (adolescent)

One adolescent reported that he went to the psychologist to receive help in maintaining contacts with friends and getting to know new friends.

Most families were still engaged in social activities with friends and relatives but organized this in a different way than before the pandemic. Examples were video calls and meeting up with each other outside whilst keeping distance. Besides this, they initiated “social bubbles” where they met with the same group of people every time to limit the number of different contacts.

“We always had the same social bubble of people who we invited over”

COLC08 (mother)

The amount of contact with others also changed during the pandemic; the more time had passed, the more people became flexible in inviting people over. Particularly after the Dutch government started the vaccination campaign, there were more contacts.

“At a certain point we were a bit looser, a bit more flexible. Because sometimes it is more important to see each other anyway”

COLC12 (mother)

The influence of the COVID-19 pandemic on social interactions with relatives also changed. In some families the contact with relatives, for example grandparents, became more intense as they took over care tasks within the family because parents had to work. In the end, some families indicated that they liked the time spent as a family without other people being present. Some children also indicated that they liked the “quieter” days.

“Sometimes I just wanted to be at home instead of being with someone else. I saw my best friend a lot. Every week every day, so every now and then I wanted some time for myself”

COLC05 (adolescent)

The important findings with regard to the wellbeing of families during the pandemic are summarized in Table 3.

## 4. Discussion

The aim of this explorative study was to describe the experiences regarding lifestyle and wellbeing of children, adolescents and their families during the COVID-19 pandemic. Our results revealed the mainly negative effects on lifestyle and wellbeing of families during the pandemic. These negative effects were even larger in adolescents and vulnerable children, e.g., those with overweight or psychosocial complaints. Besides this, we found that parents were actively involved and in general successful in maintaining a normal daily structure for their children. Diet changes were inconsistent and dependent on the availability of food. Furthermore, an increase in screen time was experienced as inevitable as both school and contact with friends took place online. Parents also used screens to keep their children occupied and external influences were necessary to keep children and adolescents active. Fortunately, the experienced negative impact on physical health in children and adolescents was limited, whereas with regard to mental health feelings of irritability, anger, loneliness and sadness were experienced in various degrees. At last, when families were asked for the most negative effect of the pandemic, most reported the decrease in social interactions.

During the COVID-19 pandemic, eating patterns of families changed, both in more and less healthy ways [20,21]. A qualitative report from the United States on family food environment during the pandemic revealed that snacking and food-seeking behavior of children increased and that some parents mentioned that their children gained weight. The parents attributed this to children being home more, increased accessibility to food and boredom [22]. Similar results and determinants were found in the current study among Dutch children and adolescents. Besides this, adolescents with overweight or severe obesity reported a further increase in their weight during the pandemic. This was in contrast to the children and adolescents who had a healthy weight, as they reported no weight gain. Notably, unhealthy eating behaviors that started in the pandemic seemed more difficult to reverse and sometimes remained, whereas the positive effects diminished. This suggests that the abovementioned unhealthy alterations in eating patterns due to the pandemic might have lasting implications for the weight of children and adolescents in the future and mainly in the children with overweight or obesity [23,24,25].

A daily structure and regular physical activity are important for the health and wellbeing of children and adolescents. During the pandemic, the ‘regular’ daily routine of the children and adolescents was lost and the usual physical activity diminished. Despite their efforts, children and adolescents seemed unable to maintain their daily routine and physical activity without help. Only the minority reported partially successful attempts to maintain this. Adolescents more often reported a decrease in their physical activity and an increase in their screen time. Our qualitative data revealed that parents felt the need to help their children in providing a daily structure and to keep them entertained and active by offering alternative activities. Due to less parental influence in the older children, this might be more successful in the younger [26]. Additionally, schools, sports clubs and public health organizations also contributed to this by organizing activities. This enlightens the importance of external factors in maintaining a healthy lifestyle of children and adolescents during the pandemic.

With regard to wellbeing, mental health decreased and the children and adolescents experienced negative emotions such as irritability, anger, loneliness and sadness in varying degrees during the pandemic. An adolescent with depressive symptoms even reported that she went from a ‘normal’ depression to a more extreme one. This is in line with previous reports that described a lower quality of life, higher anxiety levels and more mental health problems in children and adolescents during the pandemic [12,27,28]. Another nationwide study from Germany concluded that children from families with low education levels and a migration background were more at risk for developing mental health problems [5]. This suggests that the impact on mental health is even worse in the general population compared to our population, as in our study only children and adolescents from Dutch origin participated. Altogether, the pandemic seemed to place an overall burden on the wellbeing of children and adolescents. This is especially worrisome, since childhood is an important period for the social development of children and adolescents.

In previous research, the closure of schools was described as one of the most influential factors during the COVID-19 pandemic on the lifestyle and wellbeing of children and adolescents [27,28,29]. In our qualitative analysis, school was mentioned as a facilitator for a normal daily structure and normal sleeping times. Going to school also reduced snacking behavior and when children and adolescents go to school instead of having online lessons there is less screen time. Furthermore, schools provide social contact, social and cognitive development and are a safety net for children and adolescents [27]. Lastly, schools ensure physical activity by providing physical education and transport to school, which in the Netherlands is mostly done by biking or walking. Although not all participants identified the closure of schools as one of the main contributors to their changes in lifestyle and wellbeing, the closure of schools can in almost all cases be indirectly linked to these changes, whether participants were aware of this or not. Overall, extended school closures contributed to an unhealthy lifestyle and wellbeing [29].

Besides the burden, families also reported positive experiences due to the pandemic. Examples of positive experiences were increased free time, consumption of more fruit, more home cooked family dinners and spending more time with family. It was mentioned that due to the pandemic some children also became more resilient when faced with challenges. Similar to our study, an Australian study also showed that families indicated that they enjoyed spending more time together [30]. This emphasizes the differences between how families experienced the pandemic.

By combining the experiences of families during the COVID-19 pandemic, several recommendations can be made with regard to future pandemics or crises. First of all, health promotion programs should not be discontinued without offering an alternative option and should focus on parental involvement. When parents learn how to support their children during crises, they will be more likely to apply this when it is really necessary. Secondly, governmental regulations should be adjusted to the needs of children; especially, adolescents and vulnerable children (e.g., the ones with overweight or psychosocial complaints) might benefit from this. Lastly, governmental regulations to prevent the spread of COVID-19 infections need to be reviewed. Regulations must balance the threat of the pandemic with the impact the regulations have on the children’s lifestyle and wellbeing.

This study has several limitations. First, due to the design of the study, selection bias of the interviewed families should be taken into account. Families who volunteered to participate in the study were aware of the subject and might have had stronger opinions on the effect of the COVID-19 pandemic on their lifestyle and wellbeing. Besides this, the families who participated in the study are not entirely representative of the general Dutch population. For example, our study population only consisted of families of Dutch descent, whereas in the general population 26.2% of the Dutch population has a migration background [31]. Besides this, there were three children with overweight (30.0%) and two of them had severe obesity (13.3%), whereas in the general Dutch population the overweight percentage varies from 15.5–18.6% and there are only 2.9–4.3% children with severe obesity [32]. Lastly, the interviews were executed during a broad time range. Ten interviews were done in May and June 2021 and five interviews in October and November 2021. Therefore, the interviews were in different stages of the COVID-19 pandemic. At the end of May 2021, the biggest part of society opened up again, whereas in November 2021 new stricter regulations such as a maximum of four people at home applied [1]. These differences could have led to disparities in recall bias. Families who were interviewed when strict governmental regulations applied, might have experienced more negative feelings with regard to COVID-19 compared to families who were interviewed when almost no governmental regulations applied.

## 5. Conclusions

Even though children and adolescents appear to be less affected by a COVID-19 infection and most of the interviewed families came from a stable family environment, they faced impactful challenges. The families experienced in general an unhealthier lifestyle and decreased wellbeing, and the older children and the ones with overweight or psychosocial complaints were even more affected. Our qualitative data revealed that children and adolescents are not always able to cope with these challenges without help. External influences, such as the influence of caregivers, schools and public health efforts, are necessary to support them in obtaining a healthy lifestyle and wellbeing. These insights may aid in the development of preventive measures to promote a healthy lifestyle and wellbeing of children and adolescents during future pandemics or other crises. Additionally, it creates awareness of the lasting effects of the COVID-19 pandemic on lifestyle and wellbeing of children and adolescents, even after most regulations were lifted.

## Figures and Tables

**Table 1:** Characteristics of the included children, adolescents and their families.

	Children, Adolescents and Their Families, *n* = 15
Age category ^1^, no.	
Children	7
Adolescents	8
Sex, no.	
Female	9
Male	6
IOTF classification ^2^, no.	
Healthy weight	12
Overweight	1
Obese	0
Severely obese	2
Weight change during pandemic, no.	
Weight loss or unchanged	12
Weight gain	3
Family composition ^3^, no.	
Intact	14
Not intact	1
Number of children in family, no.	
One child	3
Two children	6
Three or more children	6
Working status parents ^4^, no.	
Employed	13
Unemployed	2

Data is presented as number. N = number, IOTF = International Obesity Task Force. ^1^ Children aged 4–11, adolescents aged ≥ 12 [13,14]. ^2^ According to International Obesity Task Force [15]. ^3^ Intact family means parents are in a relationship. ^4^ Employed means both parents have a job.

**Table 2:** Important findings with regard to the lifestyle of families during the COVID-19 pandemic.

	Important Findings
Daily structure	Parents actively tried to maintain their children’s daily routine as it was before the pandemic Older and vulnerable children (e.g., youth with overweight or psychosocial complaints) often reported changes in their daily structure
Diet	Diet changes were inconsistent across families and dependent on the availability of food at home, the workload of parents and boredom of the children The positive effects of the pandemic on the food behavior of families diminished as soon as life went back to normal again, whereas the unhealthy behaviors were more difficult to reverse
Screen time	The screen time of children and adolescents has inevitably increased according to the families, as school and contact with friends took place online
Physical activity	Children and adolescents needed external factors (e.g., influence of parents or organization of alternative activities) to stay active The younger children were more approachable for external factors

**Table 3:** Important findings with regard to the wellbeing of families during the COVID-19 pandemic.

	Important Findings
Physical health	Limited influence of the pandemic on the physical health of children and adolescents
Mental health	Experienced decrease in mental health of children and adolescents, especially in the older and more vulnerable children, e.g., the ones with overweight or psychosocial complaints Feelings of irritability, anger, loneliness, sadness, boredom or fear were reported by almost all children and adolescents
Social life	Loss of social contact with friends was the most negative effect of the pandemic according to the children and adolescents During the pandemic other ways to stay in touch with friends or family were sought, e.g., “social bubbles” or video calls

## Data Availability

The data presented in this study are available on request from the corresponding author. The data are not publicly available due to the nature of the interviews. It is not possible to publish the full text without compromising participant confidentiality. On request the interviews can be shared with others, leaving out information that could be traced back to individuals. For example: names, schools, employers and other personal information. The text that is left out will be explained so that it is understood what type of information is missing.

## Supplementary Materials

The following supporting information can be downloaded at: https://www.mdpi.com/article/10.3390/children9121929/s1. File S1: Interview guide semi-structured interviews; File S2: Coding tree.
