# Supplementary material for: The Impact of the COVID-19 Pandemic on Lifestyle and Wellbeing of Children, Adolescents and Their Parents: A Qualitative Study
Source: Children (Basel). 2022 Dec 8;9(12):1929. doi: 10.3390/children9121929 (PMC9776844; doi:10.3390/children9121929)
Supplement: Supplementary file 1 [file children-09-01929-s001.zip › children-2067517-supplementary.pdf]

**Supplementary files**

*File S1. Interview guide semi-structured interviews*

| *Questions on daily life and school* |
|---|
| We are curious about your hobbies… |
| What do you think of online school? |
| Can you tell us what a school day was like when the schools were completely closed and everything was online? |
| Are parents/siblings also working from home? |
| Schools are currently open again, what do you think about this? |
| Have your sleeping times changed since the pandemic? |
| If you are bored, what do you do? Did this change since the pandemic? |
| For parents: has the pandemic affected your child's daily rhythm? |

| *Questions on food and drinks* |
|---|
| What do you eat for breakfast? Lunch? Dinner? Snacks? And do you eat anything before going to bed? |
| Are the things you eat different compared to when you are at school? |
| For parents: has the pandemic affected your child's diet? If so, how? |
| Do you eat together at the dinner table at fixed times? Was that also the case before the pandemic? |
| How many days a week do you cook at home? What do you eat on other days (ready-to-eat meals, take away)? How was this before the pandemic? |
| Have you started eating more or fewer snacks since the corona pandemic? What could be the reason for this? |
| Can you explain whether you always eat when you are hungry? Do you sometimes also eat when you are not hungry? |
| How many fruit and vegetables do you eat in a day? |
| Has anything changed about this since the pandemic? |
| When you are thirsty, what do you drink? Can you tell me what you drink in a day? |
| Are you drinking more or less sugary drinks than before the pandemic? What could be the reason for this? |
| For parents: are there positive changes due to the pandemic? |

| *Questions on physical activity, sports and free time* |
|---|
| How many days a week do you play outside? |
| What do you do when you play outside? |

| |
|---|
| Where do you play outside? Do you have a garden or is there a playground nearby? Do you live on a busy street? |
| Is this different compared to before the pandemic? |
| How many hours a week do you exercise? |
| What sport do you do? Have you joined a sports club? |
| Is this different compared to before the pandemic? If so, did you miss exercising? Did you miss seeing your team? |
| Sports clubs have been temporarily closed during the lockdown. Did your sports club arrange something for this? |
| Sports clubs are now allowed to be open again, have you started exercising again? |
| Did you go on walks or bicycle rides? Has that changed since the pandemic? |
| What do you do to exercise together as a family? |
| Did you do extra things to be active, such as going on bear hunts or the toilet roll challenge? |
| Does your school give physical activity lessons? |
| How do you travel to school? By bike, car, bus or on foot? |
| How do you travel to your sports club? By bike, car, bus or on foot? |
| How many hours a day do you use screens during the week (telephone, television, game console, laptop/computer, iPad)? |
| How many hours for fun activities? How many hours for school? |
| How many hours do you use screens during the weekend? |
| What was this like before the pandemic? If it is different, do you have an idea what caused this? |
| When you play indoors, what do you prefer to do? |
| Did the pandemic bring any positive changes? |

| *Questions on physical health* |
|---|
| Do you have a lot of energy, or are you tired a lot? |
| Do you ever get sick or have a cold? |
| How often have you experienced this in the past year? |
| Did you have to visit a doctor for this? |
| If you could rate your physical health, what would you rate it? 0 is very bad, 10 is the best. |
| For parents: do you agree with this rating? Did the pandemic affect this rating? |

| *Questions on how you feel* |
|---|
| Are you happy and cheerful (is this different compared to before the pandemic)? |

| |
|---|
| Do you ever feel sad (is this different compared to before the pandemic)? |
| Do you ever feel lonely (is this different compared to before the pandemic)? |
| How is it going at school (is this different compared to before the pandemic)? |
| Do you ever get scared or feel nervous (is this different compared to before the pandemic)? Can you explain what you feel if you do? Are you or have you been afraid of corona? |
| Do you ever feel angry without knowing exactly why (is this different compared to before the pandemic)? |
| If you could rate your happiness, what would you rate it? 0 is very bad, 10 is the best… |
| For parents: do you agree with this rating? Did the pandemic affect this rating? |
| If you feel uncomfortable, does this influence the way you eat and exercise? How (for example more snacking, less exercise)? For parents: what is your vision on this matter? |

| *Questions on friend and family* |
|---|
| How many friends do you have? |
| How often did you meet up with your friends during the corona pandemic? Has this changed due to the corona pandemic and how? |
| Were you able to visit your grandparents during the pandemic? |
| Have you been able to see other family members during the pandemic, such as nieces and nephews? How many times per month? |
| What fun activities did you do during the corona pandemic? Did you do more or less fun activities before the corona pandemic? |
| How do you as a family feel about meeting other families? |

| *Questions on your expectations* |
|---|
| What do you miss most? What is the most annoying thing about corona? |
| If you needed help, was there help for you? |
| Is there something nice about corona? |
| How would you like to be helped to get through the pandemic as healthy as possible? What would you need for that? |
| What do you think will happen when everything goes back to normal again? Will things change for you or not? |
| For parents: what is the most important thing that came up during this conversation? |

| *General questions* |
|---|
| Sex? |
| Length? |

| |
|---|
| Weight? |
| Weight increase or decrease not in line with normal growth during the pandemic? |
| Length of parents? |
| Weight of parents? |
| Job of parents? Home office? |
| Social situation (parents together/divorced, living situation, brothers/sisters)? |
| School-level? |
| Nationality? |

*File S2. Coding tree*

| Theme | Category | Subcategory |
|---|---|---|
| Lifestyle | *Physical activity* | Playing outside |
| | | Challenges |
| | | Family |
| | | Sports at school |
| | | Screen time |
| | | Sports |
| | | Transport to school |
| | *Daily structure* | Daily rhythm |
| | | Sleeping hours |
| | *Diet* | Drinks |
| | | Diet |
| | | Main meals |
| | | Cooking and take away |
| | | Snacks |
| | | Boredom |
| Wellbeing | *Physical health* | COVID-19 |
| | | Energy |
| | | Illness |
| | *Mental health* | Impact of emotions |
| | | Negative emotions |
| | | Positive emotions |
| | *Social life* | Activities |
| | | Meeting up during COVID-19 |
| | | Family |
| | | Friends |
